# An Unreported Clindamycin Adverse Reaction: Wrist Monoarthritis

**Published:** 2012

**Authors:** Ahmad Alikhani, Ebrahim Salehifar

**Affiliations:** a *Department of Infectious Diseases, Razi Hospital of Ghaemshahr, North Iranian Infectious Antimicrobial Research Center, Mazandaran University of Medical Sciences, Sari, Iran. *; b *Department of Clinical Pharmacy, Thalassemia Research Center, Faculty of Pharmacy, Mazandaran University of Medical Sciences, Mazandaran, Sari, Iran.*

**Keywords:** Skull, Osteomyelitis, Clindamycin, Monoarthritis

## Abstract

Clindamycin is a lincosamide antibiotic which is approved for the treatment of *Anaerobic, Streptococcal *and *Staphylococcal *infections. There has been an increased interest in the use of clindamycin since it achieves high intracellular levels in phagocytic cells, high levels in bone and appears to have an antitoxin effect against the toxin elaborating strains of streptococci and staphylococci. Clindamycin is considered as a bacteriostatic antibiotic, while it is bactericidal against some strains of *Staphylococci, Streptococci *and *Anaerobes *such as *B. fragilis*. Its major disadvantage is its propensity to cause antibiotic-associated diarrhea. In spite of expanded use of clindamycin in bone infections, the adverse reactions of this antibiotic are minor. Polyarthritis is a rare adverse effect of this antibiotic.

In this case report, we studied a 75-year-old male patient with past history of drop attack and subdural hematoma who developed skull osteomyelitis after the surgery. After two weeks of intravenous antibiotic therapy, wound discharge was stopped and the patient was discharged from the hospital with the maintenance oral antibiotic therapy including clindamycin 300 mg q8 h, ciprofloxacin 500 mg q12 h and rifampin 600 mg fasting.

Six days after the beginning of oral antibiotics, right wrist monoarthritis was developed. It was unresponsive to nonsteroidal anti-inflammatory drug and improved after decreased doses of clindamycin. As best as we know, monoarthritis was not reported with clindamycin previously.

## Introduction

Clindamycin is a lincosamide *antibiotic *which is approved for the treatment of anaerobic, *streptococcal *and *staphylococcal *infections. There has been an increased interest in the use of clindamycin as it achieves high intracellular levels in phagocytic cells, high levels in bone, and appears to have an antitoxin effect against the toxin elaborating strains of *streptococci *and *staphylococci. *Clindamycin may potentiate the opsonization and the phagocytosis of bacteria even at the subinhibitory concentrations (1, 2).

By disrupting the bacterial protein synthesis, clindamycin causes changes in the cell wall surface which decreases the adherence of bacteria to host cells and increases the intracellular killing of organisms. Clindamycin is considered a bacteriostatic antibiotic, while it is bactericidal against some strains of staphylococci, streptococci and anaerobes such as *B. fragilis *(3).

High bioactivity is found in bile, mostly as the *N*-dimethyl metabolite; this represents a minor route of excretion and accounts for the activity assayed in feces after the parenteral administration (4, 5). The activity of clindamycin in feces persists for at least 5 days after 48 h of parenteral administration and is associated with a major reduction in the population of sensitive bacteria in the colon that lasts for up to 14 days (6). The concentration of clindamycin in bile is markedly diminished or absent when the common bile duct is obstructed (7-9).

Diarrhea has been reported in 2 to 20 percent of those receiving clindamycin. Typically, the diarrhea is mild and self-limited in nature and resolves upon discontinuation of the drug. Clindamycin has been frequently implicated in antibiotic-associated diarrhea due to *C. difficile *(10, 11). Other gastrointestinal side effects have been reported with clindamycin. These include nausea, vomiting, flatulence, metallic taste, anorexia, and esophagitis.

Maculopapular rash has been noted in up to 10 percent of patients receiving clindamycin. Other reactions such as drug fever, eosinophilia, erythema multiforme, and urticaria have also been reported. Some cases have resembled Stevens-Johnson syndrome. Although rare, cardiopulmonary arrest and hypotension have been reported with rapid intravenous infusions of clindamycin.

Injection-site pain and swelling have been reported with the use of intravenous and intramuscular clindamycin. Thrombophlebitis may occur with infusions of intravenous clindamycin. Less common adverse reactions reported with the use of clindamycin includes the elevation of liver transaminases, jaundice and polyarthritis. Hematopoietic effects such as neutropenia, leukopenia, agranulocytosis, and thrombocytopenic purpura have also been reported. Renal dysfunction associated with the use of clindamycin is rare, but may be characterized through oliguria, azotemia and proteinuria (12, 13).

## Case report

Two months ago, a 75-year-old male with past history of drop attack and subdural hematoma was treated surgically with open drainage and received cephalexin capsule q6 h. One month after the surgery, he developed wound infection and received antibiotic and surgical debridement. Due to the persistent wound discharge, infectious diseases specialist consultation was requested and skull osteomyelitis was suggested. A CT scan of his brain showed irregularities of bone border. Past medical and drug history was negative. Other findings were normochromic-normocytic anemia, negative CRP, normal WBC count and an elevated ESR equal to 50 mm/h. In wound culture, the methicillin-sensitive *Staphylococcus aureus *was grown.

Considering all clinical and paraclinical evidences compatible with skull osteomyelitis, intravenous (IV) 1 g of vancomycin q12 h plus 500 mg of oral ciprofloxacin q12 h and 600 mg of rifampin daily begun and the patient transferred to the infectious ward. The debridement of infected soft tissues and bone was performed through a neurosurgeon. After two weeks of intravenous antibiotic therapy, wound discharge was stopped and the patient was released from the hospital with the maintenance oral antibiotic therapy including clindamycin 300 mg q8h, ciprofloxacin 500 mg q12 h and rifampin 600 mg fasting.

After six days, the patient developed right wrist swelling, erythema and limitation of motion without fever, chills, night sweating, urinary problem and involvement of other joints. Except for the findings in the right wrist and arthritis without local swelling or tenderness that is compatible with tendinitis, other physical examinations were normal. The laboratory findings were as following: ESR: 31 mm/h, CRP: +1, Wright and Coombs Wright tests, rheumatoid factor and anti CCP were negative and uric acid was 4.5. BUN, Cr and U/A were normal.

The arthritis was unresponsive to a five-day course of treatment with Gelofen. After the reduction of clindamycin dosage to 150 mg q8 h, arthritis improved and triple therapy continued with clindamycin, ciprofloxacin 500 mg q12h and rifampin 600 mg fasting for three months. In follow-up visits, the wound was normal and the patient had no problem after 3 months.

## Results and Discussion

In spite of the expanded use of clindamycin in bone infections, the adverse reactions of this antibiotic are minor. Polyarthritis is the only joint adverse reaction that has been reported rarely and the monoarthritis has not been reported in articles.

In a study by Norden *et al*., clindamycin was used alone for the treatment of experimental osteomyelitis due to *Staphylococcus aureus *in rabbits. The results of a four-week treatment with clindamycin for chronic experimental staphylococcal osteomyelitis, were significantly better than those obtained with any other single agent used in prior studies and were generally as good as those with combination therapy that included rifampin (14).

Calcagno *et al. *reported that osteomyelitis of the skull (SO) is a rare condition. The infection may complicate community-acquired sinusitis, otitis, or mastoiditis, in which case, the skull base is affected most commonly. The flora typically seen in these conditions, such as *Staphylococcus pneumonia *and *H. influenzae*, tends also to be responsible for the SO. Osteomyelitis also may follow neurosurgical procedures that breach the skull, in which case, the pathogens frequently are typical cutaneous flora such as *Staphylococcus aureus *or *coagulase-negative staphylococci *(15).

Clindamycin phosphate was used in the treatment of 29 children with osteomyelitis by Rodriguez *et al*. The usual dose was 50 mg/Kg/day intravenously for approximately three weeks followed by oral clindamycin palmitate at home in a dose of 30 mg/Kg/day for an additional six weeks. *Staphylococcus aureus *was isolated in 22 of 29 cases: 96% of strains were penicillin resistant. There was prompt clinical and bacteriologic response shortly after the initiation of clindamycin therapy. Good bone penetration of the drug was observed (16).

In one report from FDA and community, 6, 211 people who took clindamycin Hcl and had side effects were studied. Common side effects, effectiveness and long term effects of the drug were included in this assay. Multiple side effects were reported in it and there was no articular adverse effect (17).

Many authors reported their clinical experience in patients with osteomyelitis (OM) of the jaw, focusing on aspects of antimicrobial resistance. *Viridans streptococci *were the most commonly isolated agents that were resistant to clindamycin. It was suggested that in patients with prior antibiotics exposure, a high percentage of infections were caused through clindamycin-resistant microorganisms; therefore, beta-lactams should be the antibiotic of choice. In penicillin-allergic cases, the new fluoroquinolones, probably in combination with rifampin and/or clindamycin, could be a promising alternative (18).

Although arthritis is an adverse reaction of clindamycin, as reported by many authors, clindamycin could be used in the treatment of arthritis and osteomyelitis (15, 16, 18, 19).

In study of Geddes *et al*, clindamycin has also been used for the treatment of septic arthritis. In a trial on fifty-four patients with acute and chronic septic arthritis and osteomyelitis, forty-seven responded satisfactorily. Adverse reactions were observed in 9 patients but all were minor (19).

Polyarthritis, has also been reported as a rare adverse effect of clindamycin in the majority of drug reaction reports (0.21%) (21). The age of people who had polyarthritis when taking clindamycin hydrochloride was greater than 60 years old (20, 21).

Ducroix-Roubertou *et al *reported an extra colonic manifestation of *C. difficile *infections. They reported the case of a monoarticular arthritis following pseudomembranous colitis. A 45-year-old man was admitted for fever and monoarthritis of the left knee, 8 days after the onset of a *C. difficile *enterocolitis associated with urethritis (22).

Although we should consider that monoarthritis may be associated with pseudomembranous colitis due to clindamycin, monoarthritis as a pure adverse effect of clindamycin was not reported until now.

## Conclusion

Monoarthritis should be considered as a rare adverse effect of clindamycin and could be managed with dose-reduction strategy.
